# Covid-19 infection in pregnant women in Dubai: a case-control study

**DOI:** 10.1186/s12884-021-04130-8

**Published:** 2021-09-28

**Authors:** Komal Sundeep Hazari, Rasha Abdeldayem, Litty Paulose, Nimmi Kurien, Zukaa Almahloul, Hozaifah Mohammad, Taghrid Faek A. Elgergawi, Maryam Alkhanbouli, Khalid Mahmoud, Atif Bashir Fazari, Amar Hassan, Riad Bayoumi

**Affiliations:** 1 Latifa Women and Children Hospital, Dubai Health Authority, Dubai, United Arab Emirates; 2 Rashid Hospital, Dubai Health Authority, Dubai, United Arab Emirates; 3 Mohammed Bin Rashid University of Medicine and Health Sciences, Dubai, United Arab Emirates; 4 College of Medicine, Mohammed Bin Rashid University of Medicine and Health Sciences, Dubai, United Arab Emirates

**Keywords:** Covid-19, High-risk pregnancy, Neonate, Pneumonia, Neonatal morbidity, Maternal morbidity, Dubai

## Abstract

**Background:**

Whilst the impact of Covid-19 infection in pregnant women has been examined, there is a scarcity of data on pregnant women in the Middle East. Thus, the aim of this study was to examine the impact of Covid-19 infection on pregnant women in the United Arab Emirates population.

**Methods:**

A case-control study was carried out to compare the clinical course and outcome of pregnancy in 79 pregnant women with Covid-19 and 85 non-pregnant women with Covid-19 admitted to Latifa Hospital in Dubai between March and June 2020.

**Results:**

Although Pregnant women presented with fewer symptoms such as fever, cough, sore throat, and shortness of breath compared to non-pregnant women; yet they ran a much more severe course of illness. On admission, 12/79 (15.2%) Vs 2/85 (2.4%) had a chest radiograph score [on a scale 1-6] of ≥3 (*p*-value = 0.0039). On discharge, 6/79 (7.6%) Vs 1/85 (1.2%) had a score ≥3 (*p*-value = 0.0438). They also had much higher levels of laboratory indicators of severity with values above reference ranges for C-Reactive Protein [(28 (38.3%) Vs 13 (17.6%)] with *p* < 0.004; and for D-dimer [32 (50.8%) Vs 3(6%)]; with *p* < 0.001. They required more ICU admissions: 10/79 (12.6%) Vs 1/85 (1.2%) with *p*=0.0036; and suffered more complications: 9/79 (11.4%) Vs 1/85 (1.2%) with *p*=0.0066; of Covid-19 infection, particularly in late pregnancy.

**Conclusions:**

Pregnant women presented with fewer Covid-19 symptoms but ran a much more severe course of illness compared to non-pregnant women with the disease. They had worse chest radiograph scores and much higher levels of laboratory indicators of disease severity. They had more ICU admissions and suffered more complications of Covid-19 infection, such as risk for miscarriage and preterm deliveries. Pregnancy with Covid-19 infection, could, therefore, be categorised as high-risk pregnancy and requires management by an obstetric and medical multidisciplinary team.

## Background

Coronavirus disease 2019 (Covid-19) is caused by the novel severe acute respiratory syndrome coronavirus 2 (SARS-CoV-2). In March 2020, the Covid-19 outbreak was declared a pandemic by the World Health Organization [1, 2]. By January 2021, more than 90 million confirmed cases had been reported globally, with a mortality rate of 2.2%. In the United Arab Emirates, a total of 233,000 cases were reported, with 710 deaths [3]. Currently, there is appreciable data available on Covid-19 infection in pregnant women in the literature worldwide. However, there is little published data on the pattern of Covid-19 infection in pregnant women and the maternal/foetal outcomes from the Middle East in general, and the United Arab Emirates in particular [4].

The maternal immune system faces challenges in establishing and maintaining tolerance to the foetus and simultaneously defending against microbial challenges. Physiological changes that occur in the respiratory and circulatory systems during pregnancy, such as an elevated diaphragm, enhanced oxygen consumption and oedema of the mucosa of the respiratory tract, reduce tolerance to hypoxia and can worsen clinical outcomes when infected with a virus. This makes the women more vulnerable to severe symptoms from viral infections, as seen in studies of the common flu, severe acute respiratory syndrome (SARS) and Middle East respiratory syndrome [5–8].

Although the mortality in the overall population was ≥ 2.6% in the 1918 influenza pandemic, it was as high as 37% among pregnant women [9, 10]. An increased risk of complications in pregnant women was also reported during the 2009 H1N1 influenza pandemic, and pregnant women were more than four times more likely to be admitted to hospital than were the general population [6]. Wong and colleagues [5] reported that 50% of pregnant women who developed SARS were admitted to an intensive care unit (ICU); around 33% required mechanical ventilation and the mortality rate was as high as 25%.

To date, the two largest studies on Covid-19 in pregnancy were carried out by the Oxford University National Perinatal Epidemiology Unit in the UK Obstetric Surveillance System [11, 12] and the US CDC National Notifiable Diseases Surveillance System [13, 14]. ‘These two studies reported the impact of Covid-19 infection in 427 pregnant women in the UK and 23,434 women in the US. Interim reports from both studies confirmed the generally mild clinical course of SARS-CoV-2 infection during pregnancy.

In the UK series, most women came from black or minority ethnic groups. They presented with mild symptoms in early pregnancy but had moderate-to-severe illness in late pregnancy. Despite this, most women had good outcomes. The disease severity in pregnant women who were hospitalised was comparable to that of the general population, with a mortality rate of 1.0% [12]. Transmission of SARS-CoV-2 to infants was uncommon. In the US, pregnant women with Covid-19 infection were more likely to be admitted to hospital and receive respiratory support than non-pregnant women, as was found in the UK-based report [14].

Data on the clinical manifestations, risk factors and maternal and perinatal outcomes of Covid-19 in pregnancy have recently been compiled in a systematic review and meta-analysis of 77 studies with over 11,432 patients by Allotey et al. [15]. The results were essentially similar to the two major studies from the UK and US.

In this study, we aimed to examine the impact of Covid-19 infection on pregnant women in the United Arab Emirates population and examine the clinical picture of this disease against the worldwide view. To this aim, we carried out a case-control study to compare the clinical course between pregnant and non-pregnant women with Covid-19, as well as to assess the outcome of pregnancy in pregnant women, in Dubai. The patients were admitted to Latifa Women and Children Hospital in Dubai between March and June 2020.

## Patients and methods

### Study design and population

The study was conducted in Latifa Women and Children Hospital, which is a 440-bed public tertiary care centre in Dubai that specialises in maternal, neonatal and paediatrics services. During the Covid-19 pandemic, most of the hospitals’ wards were converted into isolation units for infectious diseases to accommodate patients with Covid-19.

From March 21st to June 30th, 2020, 444 patients (242 males and 202 females) were admitted to Latifa Hospital with Covid-19 infection. A Questionnaire was administered to establish if they had contact with someone positive for Covid-19. Of these, all pregnant women positive for Covid-19 were enrolled in the study. They were admitted for medical or obstetric reasons or had confirmed contact with a person infected with Covid-19. All patients were tested by Reverse Transcription-Polymerase Chain Reaction (RT-PCR) using nasopharyngeal swab for respiratory tract infection by SARS-CoV-2.

Seventy-nine pregnant women with confirmed SARS-COV-2 infection were identified and included in the case arm of the study. Their mean gestational age at presentation was 26 weeks; 17 (22%) were in their first trimester, 15 (19%) were in their second trimester, and the remaining 47 (59%) were in their third trimester. A total of 85 non-pregnant women positive for Covid-19, admitted during the same period, were included in the control arm of the study. A special effort was made to best match the cases and controls in terms of demographic features and the presence of co-morbidities, such as a prior diagnosis of cardiac disease, hypertension, diabetes or respiratory disease, and any medication used.

Within the case and control arms, we further categorised patients as asymptomatic, mild, moderate, severe or critical according to the National Institutes of Health categorization of Covid-19 severity [16], which is as follows:

i. *Asymptomatic*: Patients have no symptoms but are positive for SARS-CoV-2.
ii. *Mild*: Patients have symptoms and signs of Covid-19, such as fever, cough, sore throat, malaise, headache, or muscle pain, but no shortness of breath, dyspnoea, or abnormal chest X-ray findings.
iii. *Moderate*: Patients have clinical or imaging findings suggestive of lower respiratory disease but have maintained an oxygen saturation above 93% on room air.
iv. *Severe*: Patients’ respiratory rate is above 30/min, oxygen saturation is below or equal to 93%, PaO2/FiO2 < 300 and/or pneumonic infiltrates involving more than 50% of the lung are seen.
v. *Critical*: Patients suffer respiratory failure, septic shock and/or multiple organ dysfunction, probably due to cytokine storm [17, 18].

### Data collection

Data were collected from both study arms (pregnant and non-pregnant) from the Dubai Health Authority’s unified electronic medical records system, which is called “Salama”. The information collected was categorised and included demographic data, medical and obstetric history, physical examination results, laboratory investigation results, chest radiographs, treatment protocols, and maternal and neonatal outcomes. The standardised collected data were entered into an SPSS data collecting sheet designed specifically for this study.

### Chest X-ray scoring

To determine the severity of pneumonia, a chest radiograph severity scoring system was adopted [19]. Each lung was divided into upper, middle, and lower zones, resulting in a total of 6 zones per patient. A zone was given a binary score depending on the presence or absence of opacities. The images were also classified as exhibiting ground glass opacities, reticular patterns, or consolidations, according to a recent report from Toussie et al. [19].

The lower lung zone extends from the costophrenic sulcus to the inferior hilar markings; the middle zone from the inferior hilar markings to the superior hilar markings; and the upper zone from the superior hilar markings to the apices.

### Treatment protocol

COVID-19 positive patients were treated according to the UAE National Guidelines for Clinical Management and Treatment of COVID-19 [20] and following the NIH guidelines [16]. Based on the clinical severity and after thorough assessment of the patient, the COVID-19 patients were treated with appropriate antimalarials (Chloroquine Phosphate/Hydroxychloroquine Sulfate), antivirals (Favipiravir in non-pregnant women), antiretrovirals (Lopinavir / Ritonavir), steroids, antibiotics, immunosuppressants (Tocilizumab), Interferon and prophylactic or therapeutic anti-coagulants (Table 1). Patients were considered cured after two consecutive negative (RT-PCR) nasopharyngeal swabs.

**Table 1:** The drugs and treatment regimens used in treatment of women with Covid-19 infection

Generic name	Dose	Duration
Hydroxychloroquine	Two loading doses of 400 mg followed by 200 mg twice daily	7-14 days
Chloroquine base	300 mg (base) twice daily	5-7 days
Lopinavir-Ritonavir (200mg/50mg)	400mg/100mg twice daily	5-7 days (maximum 14 days)
Favipiravir	Two loading doses of 1600 mg over 1 day followed by 600 mg twice daily	5-7 days
Tocilizumab	4-8 mg/kg (max 400 mg) followed by a second dose after 8-12 hours, and then a third dose 8-12 hours later	3 doses within 24 hours
Pegylated interferon	180 mcg, maximum of 2 doses 1 week apart	2 doses within one week
Low Molecular Weight Heparin (LMWH) [Prophylactic]	According to Body weight: 50-90kg: 40mg once daily. 91-130kg: 60mg once daily. 131-170kg: 80mg once daily.	Till clinical improvement
Low Molecular Weight Heparin (LMWH)-Therapeutic in critical Covid-19 pneumonia patients	50-90kg: 40mg twice daily 91-130kg: 60mg twice daily 131-170kg: 80mg twice daily	Till clinical improvement
Methylprednisolone	0.5-1 mg/kg in 2 divided doses	3 days in Non-ICU & 5-7 days in ICU patients.
N-acetyl cysteine	Two 1200 mg doses followed by 600 mg thrice daily	7-14 days

### Statistical analysis

Data were analysed using IBM-SPSS for Windows version 25.0 (SPSS Inc., Chicago, IL). Categorical variables are described by using proportions. Continuous variables are described by a measure of tendency and a measure of dispersion. Continuous data was tested for normality by using Kolmogorov-Smirnov test. The Mann-Whitney test and t-test were used when appropriate to compare means between continuous variable. Categorical variables were cross-tabulated to examine the independence between variables; for these variables, the χ2-square test or Fisher’s exact test were used as appropriate. A *P*-value of less than 0.05 was considered significant for all analyses.

## Results

### Demographic characteristics and comorbidities

Demographic characteristics and comorbidities of pregnant and non-pregnant women with confirmed SARS-CoV-2 infection are shown in Table 2. There was a significant difference in mean age between the Covid-19-positive pregnant women (32.7 ± 5.5 years) and Covid-19 positive non-pregnant women (34.9 ± 6.9 years); with *p*-value of 0.024. There was also a significant difference in mean BMI between pregnant women (29.4 ± 5.5 kg/m^2^) and non-pregnant women (27.6 ± 5.2 kg/m^2^); with a *p*-value of 0.033

**Table 2:** Demographic characteristics and comorbidities of pregnant and non-pregnant women with confirmed SARS-CoV-2 infection

	Pregnant (*n* = 79)	Non-pregnant (*n* = 85)	*P*-value
	[n (%)]	[n (%)]	
Age (years)^a^			
<= 29	23 (29)	21 (25)	
30-40	51 (65)	46 (54)	0.024
>= 41	5 (6)	18 (21)	
BMI (kg/m^2^)^a^			
Normal	39 (49)	57 (68)	
Overweight	37 (47)	25 (30)	0.056
Obese	3 (4)	2 (2)	
Nationality [n (%)]^a^			
UAE	37 (47)	27 (32)	
Other Nationalities	42 (53)	58 (68)	0.814
Co-morbidities [n (%)]^b^			
None	48 (61)	58 (68)	0.3503
Asthma	4(5)	4 (5)	1.000
Chronic Lung Disease	3 (4)	5 (6)	0.5596
Diabetes Mellitus	14 (17)	4 (5)	0.0136
Gestational Diabetes	3 (4)	0	0.0634
Hypertension	3 (4)	7 (8)	0.2853
Hypothyroidism	3 (4)	6 (7)	0.4034
Other disease	5 (6)	1(1)	0.0788

^a^ χ2-square test was used for age, weight, and nationality

^b^ Mann-Whitney test was used for co-morbidities

A total of 39% of the pregnant women suffered from comorbidities, as compared to 32% of non-pregnant women. Diabetes mellitus was the most common co-morbidity in the pregnant women (17%) but was only 5% in the non-pregnant women.

### Symptoms

Fever was the most common presenting symptom in both groups, followed by myalgia, sore throat, cough, and shortness of breath. Headache and runny nose were more commonly observed in the pregnant group (Table 3). The frequency of presenting symptoms in pregnant women of fever, cough, sore throat, and shortness of breath, is lower than that observed in non-pregnant women. This could be related to lower rates of direct contact, established by questionnaire, of pregnant women with individuals positive for Covid-19 (27; 34%) compared to contact of non-pregnant women with individuals positive for Covid-19 (50; 59%). However, the severity of symptoms was higher among pregnant women; and worsened as pregnancy advanced.

**Table 3:** Presenting symptoms in pregnant and non-pregnant women with confirmed SARS-CoV-2 infection

	Pregnant (*n* = 79)	Non-pregnant (*n* = 85)	*P*-value
	N (%)	N (%)	
Fever	18 (23)	47 (55)	0.005
Cough	6 (8)	17 (20)	0.02
Sore throat	8 (10)	19 (22)	0.028
Shortness of breath	6 (8)	17 (20)	0.02
Headache	10 (13)	12 (20)	0.145
Running nose	10 (13)	6 (7)	0.173
Loss of smell/taste	4 (5)	4 (5)	0.599
Loose stools	3 (4)	6 (7)	0.268
Myalgia	9 (11)	24 (28)	0.006

Data were acquired at Latifa Hospital, Dubai, from March to June 2020

### Chest radiograph scores

**Fig. 1:**
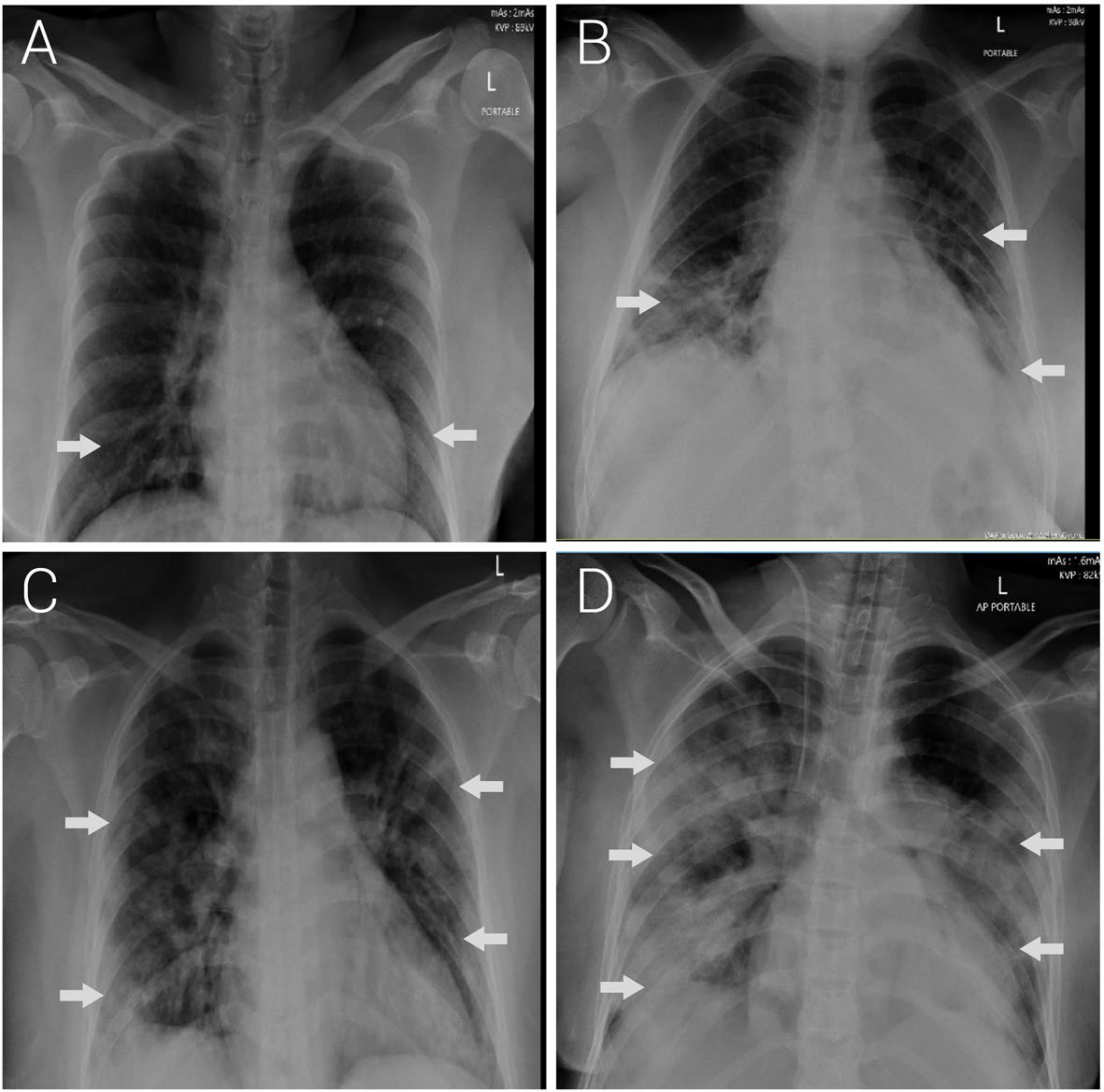
Anteroposterior radiographs depicting Covid-19 pneumonia images of pregnant women with confirmed SARS-CoV-2 infection. Images were obtained at Latifa Women and Children Hospital, Dubai, from March to June 2020. **A** Bilateral ground glass opacities involving two lung zones with associated linear opacities seen in the periphery of the right lower zone. **B** Ill-defined patchy ground glass opacities over three lung zones. **C** Bilateral multifocal peripheral lung ground glass opacity and consolidation involving four lung zones. **D** Bilateral peripheral dense consolidation with loss of lung markings in five lung zones

On admission, 12/79 (15.2%) pregnant women and only 2/85 (2.4%) non-pregnant woman had a chest radiograph score ≥3; (*p*-value = 0.0039). During their hospital stay, 11/79 (13.9%) pregnant women and 9/85 (10.6%) non-pregnant women had a score of ≥3 (*p*-value is = 0.5199). On discharge, 6/79 (7.6%) pregnant women had a score ≥3 compared to only 1/85 (1.2%) non-pregnant woman (*p*-value = 0.0438). In general, pregnant women presented with worse radiograph findings on admission and throughout their course of illness than non-pregnant women (Fig. 1).

On admission and throughout the hospital stay, two laboratory indicators of severity of Covid-19 infection; C-Reactive Protein (C-RP) and D-dimer were significantly higher in pregnant women than in non-pregnant women (Table 4). Values above reference ranges for C-RP were [(28 (38.3%) vs13 (17.6%)] with *p* < 0.004 and for D-dimer [32 (50.8%) vs 3(6%)]; *p* < 0.001. However, differences in lactate dehydrogenase [13 (18.6%) vs 8 (11.1%)]; *p*-value < 0.155 and ferritin [4 (5.8%) vs 4 (6.3%)], *p*-value 0.761, were not significantly different.

**Table 4:** Laboratory results of investigations of pregnant and non-pregnant women with confirmed SARS-CoV-2 infection

Pregnant				Non-pregnant			
Number tested/Total [≥ or ≤ cut-off value] (%)				Number tested/Total [≥ or ≤ cut-off value] (%)			
Reference range**	On admission	Peak	On discharge	Reference range**	On admission	Peak	On discharge
Hb (< 11 g/dl)	48/75 (64)	14/43 (33)	22/46 (48)	Hb (<12 g/dl)	57/84 (68)	11/23 (48)	13/24 (54)
WBC (> 16 x10^3/uL)	3/74 (4)	5/45 (11)	0/46 (28)	WBC (>11 x 10^3/uL)	1/84 (1)	2/23 (9)	1/24 (4)
Lymphocyte (<1.0 x 10^3/uL)	15/75 (20)	14/38 (37)	4/41 (10)	Lymphocyte (<0.8 x 10^3/uL)	12/81 (15)	6/18 (33)	3/18 (17)
PLT (<150 x 10^3/uL)	9/75 (12)	8/44 (18)	8/47 (17)	PLT (<150 x 10^3/uL)	4/83 (5)	2/23 (9)	4/24 (17)
PLT (>400 x 10^3/uL)	2/75 (3)	1/75 (2)	0/47 (0)	PLT (>400 x 10^3/uL)	1/83 (1)	1/23 (4)	0/24 (0)
CRP (>7 mg/L)	28/73 (38) ^**^	15/22 (68)	14/34 (41)	CRP (>7 mg/L)	13/78 (16)	8/16 (50)	4/24 (17)
PT (>14 seconds)	4/45 (9)	2/19 (11)	0/14 (0)	PT (>14 seconds)	4/72 (6)	1/7 (14.3)	0/7 (0)
PTT (>38 seconds)	17/51 (33) ^**^	9/16 (56)	6/13 (46)	PTT (>38 seconds)	42/72 (58)	6/7 (86)	5/7 (71)
Bilirubin (>1.2 mg/dL)	2/65 (3)	3 (14)	3/21 (14)	Bilirubin (>1.2 mg/dL)	1/80 (1)	0/14 (0)	1/16 (6)
SGPT (>40 iu/ L)	5/65 (8)	11/25 (44)	7/28 (25)	SGPT (>40 iu/ L)	4/81 (5)	7/16 (44)	5/17 (29)
SGOT (>40 iu/ L)	4/44 (9))	516 (31)	4/16 (25)	SGOT (>40 iu/ L)	1/32 (3)	3/4 (75)	1/5 (20)
LDH (>524 U/L)	13/70 (19)	16/30 (53)	10/29 (35)	LDH (>222 U/L)	8/72 (11)	8/16 (50)	1/13 (8)
PCT, (>0.05 ng/ml)	30/64 (47) ^*^	20/24 (83)	14/23 (61)	PCT, (>0.05 ng/ml)	19/69 (28)	7/12 (58)	6/13 (46)
Ferritin (>116 ng/ml)	4/69 (6)	8/31 (26)	6/32 (19)	Ferritin (>150 ng/ml)	4/64 (6)	2/12 (17)	3/13 (8)
D-Dimer (>0.5 mcg/ml FEU)	32/63 (51) ^**^	15/26 (58)	9/29 (31)	D-Dimer (>0.5 mcg/ml FEU)	3/50 (6)	3/11 (27)	1/10 (10)

Data were acquired at Latifa Hospital, Dubai, from March to June 2020

*Hb* Haemoglobin, *WBC* White blood count, *PLT* Platelet Count, *CRP* C-Reactive Protein, *PT* Prothrombin Time, *PTT* Partial Thromboplastin Time, *SGPT* Serum Glutamic-Pyruvic Transaminase, *SGOT* Serum Glutamic-Oxaloacetic Transaminase, *LDH* Lactate Dehydrogenase, *PCT* Procalcitonin

*p < 0.05; ** *p* < 0.01

** Separate reference ranged were used for pregnant women due to physiological changes during pregnancy

The mean white blood cell count on admission was significantly higher in pregnant women than in non-pregnant women (8.8 ± 3.6 vs. 5.8 ± 2.0; [x10^3/uL]; *p* < 0.01); while, the platelet values were not significantly different in pregnant women at peak illness than in non-pregnant women (122,000 vs. 149,000 10^3/uL); with a *p*= 0.21. There was also no significant difference between groups in liver enzymes. The Partial thromboplastin time (PTT) levels were raised in both groups at illness peak and until discharge. Haemoglobin levels were lower in pregnant women than in non-pregnant women throughout the course of the disease (peak Hb 10.2 vs. 11.2; *p* < 0.01), as is expected in pregnancy. The nadir haemoglobin level was 8.5 g/dL in pregnant women, which was similar to that of non-pregnant women (8.9 g/dL).

### Severity of Covid-19 infection

Ten (13%) of the seventy-nine pregnant women had severe or critical Covid-19 infection that required ICU admission (Table 5). Eight of these women were in their third trimester, one was in the first trimester, and one was in the second trimester. Their mean age was 32 years. Five were overweight (BMI 25-30), while five were obese (BMI ≥ 30). Two were diabetic and one had gestational diabetes. Two suffered from hypothyroidism. Four of these pregnant women (5%) required non-invasive oxygen support, while six (8%) required mechanical ventilation. One pregnant woman died of Covid-19 pneumonia due to acute respiratory distress syndrome (ARDS), multi organ failure, septic shock and disseminated intra-vascular coagulation. The remaining 69 pregnant women were asymptomatic or had mild-to-moderate symptoms.

**Table 5:** Severity of the disease in pregnant and non-pregnant women with confirmed SARS-CoV-2 infection

	Cases (*n* = 79) Number (%)	Control (*n* = 85) Number (%)	*p*-value
Asymptomatic	26 (32)	11(13)	0.0035
Mild	35 (44)	57 (67)	0.0030
Moderate	8 (10)	13 (14)	0.4335
Severe	4 (5)	2 (2)	0.2940
Critical	6 (8)	2 (2)	0.0759
Intubated and ventilated	6 (8)	1(1)	0.0290
O2 support/Non-invasive	12 (15)	3 (4)	0.0157
Complications	9 (11)	2 (2)	0.0184
Recovery time (Mean (SD))	16 (11)	15 (7)	0.3708
Death	1 (1)	0	0.3569

In the non-pregnant arm, three women required oxygen support, while only one required mechanical ventilation. All remaining 81 non-pregnant women were either asymptomatic or had mild-to-moderate symptoms. None of the non-pregnant women died. The mean recovery time for both groups ranged from 15-16 days.

### Treatment

Drugs used to treat pneumonia in women with Covid-19 are shown in Table 6. Hydroxychloroquine and Lopinavir/Ritonavir were used as first-line drugs in pregnant women, because Favipiravir is contraindicated during pregnancy. Favipiravir was used in 22 (26%) non-pregnant women. Hydroxychloroquine was used more frequently (*n* = 38) than chloroquine (*n* = 3) in pregnant women, and significantly more non-pregnant women (*n* = 16) were treated with chloroquine (*p* = 0.002). Low-molecular-weight heparin (LMWH) was used in 64 (81%) pregnant women as compared to 30 (35%) non-pregnant women (*p* < 0.001). Broad spectrum antibiotics were prescribed to 30 (38%) pregnant women for secondary infection, compared to only 11 (13%) non-pregnant women. Therapeutic LMWH was offered to 8 pregnant and 9 non-pregnant women, and prophylactic LMWH was offered to 59 pregnant and 25 non-pregnant women. Three patients received pegylated interferon, and one received Tocilizumab. Extracorporeal membrane oxygenation and plasma exchange were used in only one patient, who did not survive.

**Table 6:** Drug treatment and outcomes of the disease, in pregnant and non-pregnant women with confirmed SARS-CoV-2 infection

	Pregnant women (*n* = 79)	Non-pregnant women (*n* = 85)	*P*-value
	No. (%)	No. (%)	
Hydroxychloroquine	38 (48)	44 (52)	0.377
Favipiravir	0	22 (26)	< 0.001
Lopinavir-Ritonavir	15 (19)	15 (18)	0.492
Azithromycin	11 (14)	8 (10)	0.255
Chloroquine	3 (4)	16 (19)	0.002
LMWH	64 (81)	30 (35)	< 0.001
Methylprednisolone	12 (15)	7 (8)	0.126
Interferon	3 (4)	0	0.110
Acetylcysteine	5 (6)	4 (5)	0.454
Antibiotics	30 (30)	11 (13)	< 0.001
Vitamin C/Vitamin D	68 (86)	79 (93)	0.118
O2 support/ventilated	10 (13)	4 (5)	0.061
Complication	9 (11)	2 (2)	0.124
Recovery time			
Mean (SD)	16 (11)	15 (7)	0.453
Death	1 (1)	0	0.481

### Maternal and neonatal outcomes

The maternal and neonatal outcomes are presented in Table 7. Thirty-one pregnant women with Covid-19 infection had a mean gestational age of 36 weeks and delivered, while infected with Covid-19, between March and June 2020. In total, 12 women had preterm, while 19 had full term deliveries. Nine of the 31 pregnant women went into spontaneous vaginal delivery (SVD), while 22 had lower segment Caesarean section (LSCS). Of the 9 who had SVD, 2 were preterm and the remaining 7 were full term.

**Table 7:** Maternal and neonatal outcomes of 31 pregnant women who delivered while infected with confirmed SARS-CoV-2

***Maternal outcome***	
Total deliveries	31
Gestational age at presentation (weeks), Mean (SD)	26 (11%)
Twin pregnancy	2
Preterm deliveries	12 (38%)
Spontaneous labour and normal delivery	9 (29%)
Elective LSCS	9 (29%)
Emergency LSCS	13 (42%)
Total neonates	31
LSCS for obstetric reasons	16
LSCS for Covid-19 pneumonia	6
Spontaneous first-trimester miscarriages	4
Ruptured ectopic pregnancy	2
***Neonatal outcome***	
Birth weight (kg), mean (SD)	2.8 (± 0.7)
Apgar 1 min mean (SD)	8
Apgar 5 min	9
Covid-19-positive	2
Covid-19-negative	29
Twin delivery	1
Intra uterine foetal death	1
Fever	2
Jaundice	12
***Neonatal ICU admission reason***	
Jaundice	6
Respiratory distress	2
Prematurity	2
Mother in the ICU	1
Elevated inflammatory markers	1
NICU total admissions	12

Of the 22 women who had LSCS; 10 were preterm while 12 were full term. Thirteen out of those 22, had emergency LSCS; 6 for Covid-19 pneumonia and 7 for obstetric reasons, including 2 for severe pre-eclampsia. Of the 6 emergency LSCS performed due to severe Covid-19 pneumonia, only 2 received betamethasone for foetal lung maturity.

The most frequent Covid-19 complications observed during hospital stay and on discharge in the 79 pregnant women were, sepsis (*n* = 3), acute renal failure (*n* = 3) and ARDS (*n* = 5). One woman had post-partum haemorrhage after LSCS. Six critically ill pregnant women were in their third trimester; of these, 5 women developed what appeared to be a severe cytokine storm and had emergency LSCS for hypoxemic respiratory failure or foetal distress. They were intubated and ventilated throughout and after surgery. One woman had severe hypoxemic respiratory failure and was intubated and ventilated 2 days postoperatively.

One of the critically ill pregnant women died. At 32 gestational weeks, she presented with cyanosis, was diagnosed with severe cytokine storm, intubated, and taken for emergency LSCS. Postoperatively, she had severe ARDS, disseminated intra-vascular coagulation and septic shock, and could not be revived.

Of the remaining 48 pregnant women who recovered from Covid-19 infection and discharged from hospital, only 35 could be followed-up. Thirteen were lost to follow-up because they were either visitors to Dubai or delivered in other private health institutions. Of those 35 women with post-Covid-19 infection records, 1 patient had first trimester miscarriage, 27 had term deliveries and 7 had preterm deliveries, 1 was spontaneous for twin pregnancy. Of the 27 term deliveries, 14 were performed by LSCS: 6 had electives and 8 had emergency LSCS. The remaining 20 had normal vaginal deliveries. One pregnancy was terminated due to gastroschisis that precipitated neonatal death.

The average neonatal birth weight of the 31 neonates born during mothers Covid-19 infection, was 2.8 kg (± 0.7), with a mean Apgar Score at 1 minute of 8, and at 5 minutes of 9. Two neonates were positive for Covid-19; both were born by vaginal delivery. The first neonate was negative at birth, tested positive at 36 hours, and was negative on day 3. The second neonate was negative at birth, tested positive at 24 hours, and was negative on day 2. Both neonates remained asymptomatic throughout.

## Summary of results

On admission, most pregnant women (69/79; 87%) were either asymptomatic or suffered mild respiratory symptoms. The main symptoms, observed were fever, cough, headache, runny nose, and myalgia. In early pregnancy four women had spontaneous miscarriages and 2 had ectopic pregnancies. However, severe Covid-19 illness, ICU admission, intubation and complications were observed in late pregnancy in 10/79 (13%) women with prior comorbidities. During the study period, 31/79 women delivered: 22 (70%) had a lower segment Caesarean section, 16 for obstetric reasons and 6 for severe Covid-19 pneumonia; and 12 had a preterm delivery. Postoperatively, none showed significant improvement on account of early delivery. Sepsis, acute renal failure, and ARDS were the most common complications observed.

The neonatal outcome was comparable to that of the general obstetric population, even though 12/31 (38%) were preterm and had to be admitted to the neonatal ICU. Two neonates, born by vaginal delivery, tested positive for Covid-19 after delivery. Both were asymptomatic and tests were negative within 72 hours. It was not possible to ascertain whether vertical transmission occurred *in utero*.

## Discussion

During the first wave of the Covid-19 pandemic in the UAE, from March to June 2020, Latifa Hospital received 444 Covid-19-positive patients, including 79 pregnant women. Sero-positivity revealed a Covid-19 prevalence of 18%. In this case-control study, we compared this group of women with a group of non-pregnant Covid-19-positive women. Most of the pregnant women were UAE nationals or expatriates of Asian ethnic origin. Two thirds were in their third trimester of pregnancy. A third had comorbidities, such as obesity, diabetes mellitus and hypertension. The prevalence of Covid-19 infection and comorbidities were similar to those reported in China [21, 22], the UK [12], the US [14] and in systematic reviews using global data [15, 23–25].

On admission, pregnant women suffered mild respiratory symptoms, whereas non-pregnant women were more symptomatic. In both groups, the main symptoms were fever, cough, headache, runny nose and myalgia. However, 10/79 (13%) pregnant women, including 8 in their third trimester, had a severe or critical Covid-19 infection that required ICU admission. Four women required non-invasive oxygen support, while six required mechanical ventilation. In contrast, only 4/85 (5%) non-pregnant women had severe illness, with only 1 woman requiring mechanical ventilation. Our findings thus indicate that Covid-19 is more severe in late pregnancy in women with comorbidities; this supports previous findings of more ICU admissions, intubations, preterm deliveries and complications in women in late pregnancy with comorbidities [12, 14, 22].

During the study period, 31/79 women delivered; of these, 22 (70%) had LSCS – 16 for obstetric reasons and 6 for severe Covid-19 pneumonia. Twelve women had a preterm delivery. Patients were prescribed hydroxychloroquine, steroids and antibiotics according to the UAE national guidelines. In most pregnant women, prophylactic thromboprophylaxis was also used. Therapeutic LMWH was administered only in serious and critical cases. Sepsis, acute renal failure and ARDS were the most common complications observed in pregnant women.

The finding that severe Covid-19 illness common in late pregnancy, particularly in women with comorbidities, has also been observed in previous studies [15]. The six pregnant women who suffered unremitting fever, cytopenia, hyperferritinemia, increased CRP, increased D-dimer and high X-ray scores appeared to be engulfed in a typical cytokine storm, which has been described previously in pregnant women with severe Covid-19 pneumonia [17, 18]. All these women delivered by Caesarean section due to severe maternal hypoxemia that required mechanical ventilation. However, postoperatively, none of these women showed a significant improvement in their condition on account of early delivery. On the contrary, one woman had a rather prolonged course of assisted ventilation. There was also one postpartum maternal death in a patient who underwent a Caesarean section immediately on admission. She had a prolonged course of assisted ventilation and eventually succumbed to complications of severe ARDS and septic shock. It is unclear whether the decision to perform early LSCS without adequately controlling the cytokine storm or stabilising the patient could have contributed to her death. It is possible that the patient’s surgical trauma aggravated the cytokine response [17, 18].

In the current study, the neonatal outcome was comparable to the general obstetric population, even though 12/31 (38%) were preterm and had to be admitted to the neonatal ICU. There was no incidence of severe neonatal hypoxia or neonatal death, and all babies were discharged in good condition. Two neonates born by vaginal delivery were swab-positive for Covid-19 24 hours after delivery. Both were asymptomatic and tested negative within 72 hours. It was not possible to ascertain whether vertical transmission occurred *in utero*, during delivery via an infected birth canal, or postpartum via respiratory droplets, skin-to-skin contact or breast feeding. We did not perform tests on blood, amniotic fluid, breast milk or placental tissue; using RT-PCR. Only one intra-uterine foetal death was recorded in the current series.

The case-control study design has offered certain strengths to this study. It confirmed evidence for an association between exposure to Covod-19 and the disease. It also looked at multiple risk factors explaining severity of the disease. Pregnant women were almost equally represented in all three trimesters of pregnancy, which facilitated a comparison of the effects of Covid-19 infection at different gestational periods. Thirty-one (40%) patients delivered while in hospital, which allowed us to assess the pregnancy outcomes. However, our study is limited by the small number of enlisted patients. Furthermore, we could not obtain laboratory confirmation of whether patients with severe illness suffered from a cytokine storm; no blood cytokines were measured. Like others, the management of patients in our study was limited by our limited experience early on during the pandemic [15, 23, 24]. Methylprednisolone was used to treat the Covid-19 infection in few patients, since our study was completed prior to the evidence presented for a greater efficacy of dexamethasone by the RECOVERY Study [26]. Similarly, chloroquine and hydroxychloroquine were used in almost half of our patients, before evidence of their ineffective role in the treatment of Covid-19 infection was presented by the WHO [27]. Finally, we could not follow up patients to observe the long-term effects of Covid-19 on both mother and baby, as patients were admitted during the emergency of the pandemic and did not attend further clinics.

## Conclusions

Pregnant women presented with fewer Covid-19 symptoms such as fever, cough, sore throat, and shortness of breath compared to non-pregnant women; but they ran a much more severe course of illness. On both admission and discharge they had worse chest radiograph scores than non-pregnant women and much higher levels of laboratory indicators of disease severity. They had more ICU admissions and suffered more complications of Covid-19 infection, such as risk for miscarriage and preterm deliveries. Pregnancy with Covid-19 infection, could, therefore, be categorised as high-risk pregnancy and requires management by an obstetric and medical multidisciplinary team.

## Data Availability

The datasets generated and/or analyzed during the current study are not publicly available due as the constitute part of the confidential patients records that Dubai Health Authority will not allow investigators to disclose. Anonymized summaries in the form of database, figures and tables are available from the corresponding author on reasonable request.

## Abbreviations

ARDS: Acute respiratory distress syndrome
BMI: Body mass index
Covid-19: Coronavirus disease 2019
ICU: Intensive care unit
LSCS: Lower segment Caesarean section
LMWH: Low-molecular-weight heparin
RT-PCR: Reverse transcription- Polymerase Chain Reaction
SARS: Severe acute respiratory syndrome
SARS-CoV-2: Severe acute respiratory syndrome coronavirus 2
UAE: United Arab Emirates
